# The Dilemma of Reconstructive Material Choice for Orbital Floor Fracture: A Narrative Review

**DOI:** 10.3390/medicines9010006

**Published:** 2022-01-13

**Authors:** Akash Sivam, Natalie Enninghorst

**Affiliations:** 1 Oral and Maxillofacial Surgery Department, Royal Hobart Hospital, Hobart, TAS 7000, Australia; 2 School of Medicine and Public Health, Faculty of Health and Medicine, University of Newcastle, Callaghan, NSW 2308, Australia; Natalie.Enninghorst@newcastle.edu.au; 3 Royal Newcastle Centre, John Hunter Hospital, Newcastle, NSW 2310, Australia

**Keywords:** orbit, orbital fracture, orbital reconstruction, orbital implants, biomaterials, complications

## Abstract

The aim of this study is to present a narrative review of the properties of materials currently used for orbital floor reconstruction. Orbital floor fractures, due to their complex anatomy, physiology, and aesthetic concerns, pose complexities regarding management. Since the 1950s, a myriad of materials has been used to reconstruct orbital floor fractures. This narrative review synthesises the findings of literature retrieved from search of PubMed, Web of Science, and Google Scholar databases. This narrative review was conducted of 66 studies on reconstructive materials. Ideal material properties are that they are resorbable, osteoconductive, resistant to infection, minimally reactive, do not induce capsule formation, allow for bony ingrowth, are cheap, and readily available. Autologous implants provide reliable, lifelong, and biocompatible material choices. Allogenic materials pose a threat of catastrophic disease transmission. Newer alloplastic materials have gained popularity. Consideration must be made when deliberating the use of permanent alloplastic materials that are a foreign body with potential body interactions, or the use of resorbable alloplastic materials failing to provide adequate support for orbital contents. It is vital that surgeons have an appropriate knowledge of materials so that they are used appropriately and reduce the risks of complications.

## 1. Introduction

Orbital floor fractures were first recognised and described in 1844 by MacKenzie and Lang [1]. In 1957, Smith and Regan described orbital fractures as fractures resulting from a sudden increase in hydraulic pressure [2]. This impact is transmitted to periocular structures, resulting in pressure to the orbit that fractures the orbital floor, commonly in the posteromedial region, parallel to the infraorbital nerve where the bone is the thinnest [2,3]. This blow can be directly to the globe or to the inferior orbital rim, causing the floor to buckle [2,4]. Today, orbital fractures are frequently a result of facial trauma by motor vehicle accidents, assault, work and falls and account for 10–25% of facial fractures [5].

Symptoms are commonly periorbital ecchymosis, oedema, enophthalmos, diplopia due to restricted extraocular muscles, infraorbital paraesthesia, blurred vision, and subconjunctival haemorrhage. Less commonly blindness, globe injury, and lacrimal system injury can be identified [6,7,8,9,10]. Despite their frequency treatment is often complicated due to complex anatomy, physiology and aesthetic concerns [11,12]. Even with proper surgical technique, successful anatomical reconstruction and appropriate follow-up complications such as enophthalmos, diplopia resulting from extraocular muscle dysfunction, and infraorbital nerve hypesthesia are frequently seen during long-term follow-up [11,13].

There are three factors that impacts repair or orbital wall fractures, management, timing, and material. The management of orbital blowout fractures has long been controversial and is evolving [13,14]. There have been numerous studies investigating the timing and surgical approach. Current indications to operate are largely based on a defect size of 2 cm^2^, enophthalmos, entrapment, persistent diplopia, and radiographic evidence of fracture. Traditionally, early aggressive surgical repair, within 14 days, has been recommended and has been shown to be more effective than secondary reconstructive procedures [12,13,14,15]. Within 14 days, there is some resolution of soft tissue oedema that can improve exposure and facilitate dissection [3]. However, evidence of entrapment of extraocular muscles requires urgent reduction of periorbital soft tissues and orbital floor reconstruction [12].

Approaches used to repair orbital floor fractures include transconjunctival, subciliary, mid-lower eyelid, infraorbital, and endoscopic transantral approaches [16]. All these approaches have been proven to allow good exposure and adequate repair of orbital wall fractures. Aside from timing and approach, a third factor for the management of orbital wall fractures are the materials used. Many reconstruction materials for orbital blowout fractures have been described in the literature, including autologous bone transplants (split cranial bone, cartilage, bone fragment, dermal fat, rib), allogenic (human dura matter, lyophilised cartilage, banked bone, fascia lata, heterogenic bovine bone graft) and alloplastic material (silastic tantalum, stainless steel, vitallium, titanium, polymethylmethacrylate, polyvinyl sponge, polyurethane, polyethylene, Teflon, hydroxyapatite, gelfoam, gelfilm, supramid) [3,17]. In the late 19th century, surgical repair of orbital floor fractures were reported using stainless steel wires and antral bone fragments [7,8]. Since the 1950s, reconstruction of the orbital floor using bone substitutes and alloplastic materials has been used [9,10].

Regardless of approach or materials, the goal of treatment remains the same. Unlike other facial fractures, the goal is not typically to achieve bone healing, rather the goal is to reconstruct the defect to the normal anatomical relations of the internal orbit while avoiding complications of the procedure or implant [18].

Surgeons use materials they believe will give the best results with the lowest complication rates. Studies, however, do not consider the fact that the surgeon or surgical centre has developed expertise using a given material; when considering the choice of material, objective analysis regarding the advantages and disadvantages must be considered.

Despite there being a large body of literature that describes the repair of orbital floor fractures using autogenic, allogenic or alloplastic materials, the ideal reconstructive material for orbital floor fractures is unresolved, and remains debated. This can be attributed to a vast majority of studies presenting a comparison between one or two materials in one surgical centre, while often not acknowledging any bias of results due to the experience of surgeons/treatment centres.

There are very few studies that present in one source a comprehensive review and comparison of an exhaustive list of materials’ properties and complications.

This narrative review will analyse the literature regarding the use of materials widely used in orbital floor repair. It will assess the properties, advantages and disadvantages of materials and aim to identify areas of further research.

Therefore, the importance and value of this study is that it will provide surgeons with not only the complication rates of certain materials but the scientific background and understanding of material properties, allowing surgeons to make informed decisions regarding material choice based on scientific evidence.

## 2. Materials and Methods

### 2.1. Information Sources

The search was performed on PubMed (Medline), Web of Science and Google Scholar to obtain evidence supporting materials used for orbital floor reconstruction until January 2021. There was no restriction of language. Search keywords including orbital floor/blowout fractures, complications and the various materials used for repair, were used in the search. Eligible studies were also manually scanned to identify additional studies for inclusion. The search was narrowed to studies on scientific, physical and mechanical properties of implant materials and those investigating materials with outcome evaluations following clinical use; however, to expand the number of eligible articles, no filter was used in the search.

### 2.2. Search Strategy

A broad-based search was implemented using the text words and themes. The keywords were “orbital fracture”, “orbital blowout”, “orbital reconstruction”, “complications”, “autologous bone”, “calvarium”, “calvarial graft”, “autologous cartilage”, “autogenic lyophilized dura”, “titanium mesh”, “medpore”, “porous polyethylene”, “bioactive glass”, “allogenic”, “silicone”, “teflon”, “polyglycolic acid”, “polyglactin” and “polydioxanone”.

### 2.3. Inclusion Criteria

Inclusion criteria required studies to (1) pertain to orbital floor fractures, (2) evaluate 1 or more biomaterial for orbital floor repair, (3) report immediate and follow-up outcome measures for comparison, and (4) provide a detailed description of scientific and physical properties of biomaterial.

### 2.4. Exclusion Criteria

Studies related to surgery but not related to orbital floor fracture or orbital floor reconstruction, not relevant to implant materials, and studies that did not provide detailed description of biomaterial were excluded.

### 2.5. Data Collection and Analysis

Studies were read by the authors and information pertaining to orbital fractures, type of reconstructive materials used, properties of materials, patients and complications were gathered. Analysis was undertaken by thematical analysis of reconstructive material properties, suitability of materials, and complications.

## 3. Results

### 3.1. Study Selection

**Figure 1:**
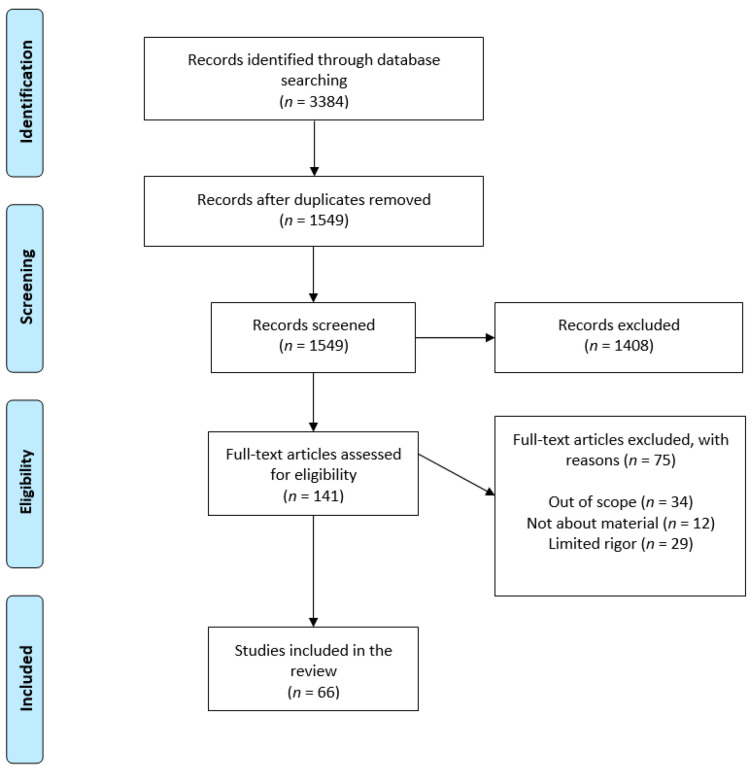
PRISMA flowchart of study selection.

All obtained studies were exported to Endnote software and were verified to remove duplicates. Authors independently screened the search results and identified studies that were potentially relevant based on title and abstract. Relevant studies were read in full and selected according to inclusion criteria. The search conducted in different electronic databases identified 3384 articles. A total of 1549 articles were short-listed after the removal of duplicates. After screening titles and abstracts, 141 studies were assessed for eligibility and 66 studies were included in the narrative review. The detail of the search is presented as PRISMA chart in Figure 1.

### 3.2. Study Characteristics

This narrative review includes 66 studies on orbital floor fractures. These studies provided data regarding the properties of materials used in orbital floor repair and the advantages and disadvantages of various materials. A total of 39 studies reported the type and number of postoperative complications associated with orbital floor repair and the remaining 27 studies discussed scientific and mechanical properties of materials used in orbital floor reconstruction. In total complications relating to 3870 patients treated for orbital floor repair with various implant materials were reported in the studies. Studies that reported postoperative complications were assigned a level of evidence according to the American Society of Plastic Surgeons Evidence Rating Scale for Therapeutic Studies (Table 1).

Table 2 presents details of 39 studies in terms of level of study, type of study, implant material and follow-up period. A total of 37 (95%) studies were level III studies and 2 (5%) were level IV studies. Of the 39 studies, 9 (23%) were prospective studies, 7 (18%) were retrospective studies and retrospective cohort studies, respectively, 4 (10%) were retrospective reviews, comparative studies, case series, respectively, 2 (5%) were case reports, and 1 (3%) was comprehensive review and follow-up study, respectively.

A number of studies have used more than one type of material, but the studies were predominantly separated into three groups, namely autologous graft materials (14 studies), allogenic graft materials (13 studies), and alloplastic graft materials (12 studies).

### 3.3. Characteristics of Materials

The goal of orbital wall reconstruction is to restore the normal anatomical relations of the internal orbit. Materials used can be divided into autologous grafts, allogenic materials, porous alloplastic, non-porous alloplastic and resorbable alloplastic materials. The ideal material has physical properties that most closely replicate those of the tissue it replaces [55]. For generic biomaterials, they should be chemically inert, biocompatible, nonallergenic and noncarcinogenic. If alloplastic, it should be cost-effective and capable of sterilization without deterioration of its chemical properties. They should be easily manipulated and adapted in the operating room and retain their form. Materials should allow for fixation to host bone with screws, wire, suture, or adhesive [6,55]; it should not potentiate the growth of microorganisms or resorption of underlying bone or distortion of adjacent structures [56]. The ideal material should be radiopaque for radiographic evaluation [48,50]. It should be easily removed if needed. It should be permanently accepted by the body, or completely resorbed with replacement of host bone [48,50,56,57].

While these characteristics are important, the long-term biocompatibility of materials depends on the relationship between the host and implant. Alloplastic materials may initiate six different biologic reactions: immediate inflammation with early rejection, delayed rejection, fibrous encapsulation, incomplete encapsulation with ongoing cellular reaction, slow resorption, and incorporation [55].

The initial cellular reaction to implanted materials is an acute inflammatory reaction, with polymorphonuclear leukocytes. Lymphocytes and macrophages then migrate to the area and attempt to phagocytise the foreign material. A chronic inflammatory reaction ensues as the material is unable to be phagocytised. Granulation tissue forms and a connective tissue sheath is formed to isolate the implant from the body’s immune response, making the implant well tolerated by the body [58].

The relationship between the body and the implant can be altered by several factors, such as chemical, mechanical, geometric, and physical factors [28,49,59,60]. Before the current implantable alloys, chemical factors would pose problems by corroding implanted metals. These factors have again become of concern in regard to resorbable materials. Resorbable materials undergo breakdown reactions and therefore have the potential to cause a host’s reaction to the breakdown products. Mechanical factors include chronic movement of the implant, discontinuity of the surrounding capsule, and ongoing trauma. These factors can lead to exposure of the implant that will practically never heal over [61]. Geometric and physical factors include size, shape, and physical form that can increase the host response to a certain material [49,59,60]. Porous materials have an increased microscopic adherence of collagen fibrils and capillaries into the pores that allow for decreased capsular contracture and long-term immobility [47]. Small increases in host reaction can affect its longevity. Optimal soft tissue capability is characterised by a limited inflammatory reaction with a thin fibrous encapsulation or mesenchymal ingrowth with minimal macrophage activity [59,62,63]. Therefore, new materials have the goal of incorporating into the host tissue and not isolating from them.

### 3.4. Autologous Materials

Autologous tissues were the first materials used to reconstruct the orbit and have long been considered the standard treatment for orbital fracture repair [64]. This requires an adequate amount of autologous material (e.g., bone) that is then shaped and inserted to provide rigid structural support to reconstruct the defect. However, they require a second operative donor site, commonly; split calvarial bone, rib, maxillary wall, mandibular symphysis, iliac crest, antral bone or coronoid process. This can increase operative time and morbidity [65,66,67]. The graft can then be placed as onlay grafts, fixed with a plate and screw, fixed with a lag screw or fixed in conjunction with an alloplastic material such as titanium mesh [33,67,68,69,70].

#### 3.4.1. Autologous Bone

The advantage of autologous bone is its inherent strength, rigidity, and vascularisation potential. As the graft material is incorporated as living tissue it does not elicit an immune response against the graft, therefore, demonstrating a relative resistance to infections, extrusion, capsule formation and ocular tethering [65]; however, the use of autologous bone is associated with less favourable outcomes such as limited ability to contour bone, variable graft resorption and donor site morbidity.

Bone is rigid so poses difficulty when contouring and has the propensity to break if moulded beyond its capacity [71]. It has been postulated that the accuracy of reconstruction is better with titanium mesh in comparison to bone grafts [29]. Bone is also of limited quantity and may not be able to be used as the sole material for large defects or fractures involving multiple walls and disruption of the bony buttresses [31,54].

The variable resorption and potential for late-occurring enophthalmos is a major concern regarding bone grafts [55]. Bone resorption occurs to a certain degree over time. Various methods have been identified to reduce the degree of resorption. The literature shows that there is up to 75% resorption for endochondral bone and 20–30% for membranous bone grafts [63,64]. Therefore, membranous bone has been shown to maintain a greater volume of the original graft. Ozaki and Buchman [68] demonstrated that resorption is not due to the embryonic origin of the bone graft, but a result of the microarchitecture. They showed cortical bone is more resistant to resorption than cancellous bone regardless of embryonic origin [68]. An alternative method to decrease resorption is to rigidly fixate the graft under mobile tissue, which promotes ingrowth of the surrounding tissue and vascularisation [72,73].

Another issue with autologous bone grafts relates to harvesting bone from a different donor site. This increases operating time and therefore time under anaesthesia, as well as donor site morbidity [65]. General risks include infection, haematoma, seroma, neurovascular injury, use of drains, increase postoperative recovery time and pain, bony defect and additional scarring. Additionally, certain donor sites are associated with site-specific risks. Rib grafts are associated with pneumothorax and split calvarial bone grafts are associated with dural tears, subarachnoid haemorrhage and intracerebral haematomas [74,75].

Calvarial bone is the preferred choice for autologous bone grafts. It is located in the same operative field, has a high volume of cortical bone, is sufficient for multiple grafts, can be easily used in conjunction with rigid fixation, and is available in sufficient amounts for children [50,70]. Iliac crest and ribs provide large quantities of bone and are relatively easier to contour than cranial bone, however, due to different microarchitectures are prone to greater resorption, have the potential for increased morbidity, and require a second operative site [20,23,38]. Alternative graft sources such as the anterior maxillary wall, ramus, and lingual cortex have been described anecdotally. Their advantages are ease of access and reduced donor site morbidity [43,75]; however, the literature is limited in quantity [76].

#### 3.4.2. Autologous Cartilage

Cartilage from the nasal septum, ribs or ear is commonly used as donor tissue for orbital floor reconstruction [26,39,65]. The benefits include its ease of harvest and contour, adequate strength, reduced donor site morbidity and reduced host immune-related complications. There is also evidence of less resorption at follow-up and the potential for cartilage grafts to calcify over time [26,63,77].

The main sources of cartilage are nasal septum and conchal cartilage [26,39]. Chowdhury and Krause [65] argue that septal cartilage resists warping and that conchal cartilage can be used for small defects and has a natural curvature that fits well in the orbital floor; however, other studies highlight cartilage has the tendency to return to its previous shape unless maintained in shape for several months, which is difficult to accomplish in the internal orbit, and that contouring cartilage will change the intrinsic tensile and extrinsic expansile forces causing a distortion of shape and therefore delayed complications [78]. Patient selection is also important; patients must be free of nasal symptoms, have no previous nasal surgery, no nasal septum deviation or spurs [40]. Autologous cartilage provides unique benefits but due to limited availability, should be used for small defects in select few patients.

### 3.5. Allogenic Materials

Allogenic materials include allografts, homografts and xenografts. They contain no living cells but possess the osteoinductive and/or osteoconductive properties, and incorporate into the host tissue and provide a structural framework for ingrowth of host tissues. The advantage over autologous grafts is the lack of donor site morbidity, decrease operating time, opportunity to prefabricate the graft and the abundance of supply [55].

Commonly used materials are human dura matter, lyophilized cartilage, banked bone, fascia lata and heterogenic bovine bone graft [51,79]. In two studies, lyophilized dura has demonstrated no infections or extrusion but has been associated with enophthalmos rates of 5.4–20% [30,51]. Demineralised and bovine heterologous bone grafts have been reported in two independent studies to have no graft related complications, incompatibility, inflammation, or infection [79,80].

Despite their positives, two main disadvantages of allogenic grafts exist. First, they have a higher resorption rate in comparison to autologous grafts. Second, the chance of transmission of infectious diseases such as HIV and hepatitis C if grafts are taken from a human donor [65,81,82]. Furthermore, there is a risk of transmission of Creutzfekdt-Jacob disease when grafts are taken from cadaveric dura [83,84].

### 3.6. Alloplastic Materials

A variety of alloplastic materials have been developed and gained popularity for the reconstruction of the internal orbit due to their ease of use and reduced surgical morbidity. Generally, these materials eliminate the need for a donor site, decrease operative time and are readily available. Disadvantages are that they are a foreign body, can elicit host reaction to the materials, and require removal of the implant if complications arise. The major subgroups are permanent materials such as metallic and non-metallic that confer a lifelong risk of complications and resorbable materials that are immune to late-occurring complications.

### 3.7. Permanent Alloplastic Materials

#### 3.7.1. Titanium

Titanium is a metallic alloplast that is rigid and malleable, making it an ideal material for reconstructing large defects requiring structural rigidity and strength. Titanium is thin, easy to contour, easily stabilised, maintains its shape, can compensate for volume when contoured without the potential for resorption, can osteointegrate and produce fewer artefacts when visualising on postoperative CT [85,86].

When comparing titanium plates and mesh versus autologous bone grafts, titanium mesh has been shown to provide better overall reconstruction with no significant complications relating to the implant material [29,57]. Sargent and Fulks [87] reviewed 54 patients who underwent repair with Vitallium mesh without bone grafts and reported excellent results with no postoperative infections or need for removal.

Disadvantages include risk of extrusion, infection and damage to soft tissues in repeat trauma. Removal of titanium has been difficult due to fibrous ingrowth and the possibility of osteointegration [86]. Lee and Nunery [41], who reviewed 10 patients repaired with titanium mesh, were presented with orbital adherence syndrome raising concerns of adhesions resulting in ocular muscle restrictions. They found that six presented with cicatricial eyelid retraction and nine with extraocular motility restriction, resulting in diplopia. Of the nine patients with diplopia, all were resolved with removal of the titanium mesh and replacement with 0.4 mm nylon implant.

Rubin and Yaremchuck [48] performed a comprehensive literature review that demonstrated 69 patients treated with titanium mesh reported no complications. However, it was noted that in 4 studies totaling 92 patients, there was an infection rate of 4.4% for metal plates, and 3.3% of implants required removal at follow-up of 6 months to 3 years [48].

#### 3.7.2. Porous Polyethylene

Porous polyethylene, available as Medpore, has been available since 1985; it is a perforated implant material that facilitates ingrowth and therefore reduced the foreign body reactions and capsule formation associated complications. Studies have demonstrated tissue ingrowth and the formation of the mucosal lining. This minimises capsule formation and therefore minimises host’s immune response and implant failure [71].

Romano et al. [47] reviewed 140 patients with facial fractures, 128 of whom had implants placed in the orbit. They reported ease of use, soft tissue ingrowth and no soft tissue adherence complications or extrusion. They found one case of infection resulting in removal of the implant. Similarly, Lupi et al. [44] used porous polyethylene in 32 patients, finding no implant migration, extrusion, or enophthalmos. However, diplopia persisted in 2 patients at 6 months.

Aside from generic disadvantages of alloplasts, porous polyethylene is not radiodense and is difficult to visualise on postoperative CT. Despite this, Wang et al. [52] suggested porous polyethylene and titanium mesh preferable to autologous bone because of decreased operative time and donor site morbidity.

#### 3.7.3. Bioactive Glass

Bioactive glass is a biocompatible material that causes minimal inflammatory response. It is a synthetic material available as blocks or small granules that produces strong chemical bonds, is osteoconductive, and eliminates the need for a donor site [19,35]. In a study of 28 patients with orbital wall fractures, 14 treated with bioactive glass and 14 treated with cartilage, no bioactive glass implants showed implant-related complications. In comparison to the cartilage group that had three cases of diplopia, and two cases of infraorbital nerve paraesthesia [19]. The main advantage is the ease of use, but the material has limited mechanical qualities. It is brittle and rigid and therefore difficult to mold, shape, contour and stabilise as overtightening screws will lead to fracture of the implant; it is therefore rarely used [88].

#### 3.7.4. Silicone

Silicone is cheap, flexible, easy to handle and provides good structural support; however, there are numerous studies that report significant complications even up to 20 years post-operatively. Laboratory studies show that silicone is more prone to fibrous capsule formation and poor incorporation at a cellular level [36,43,46,49,89,90]. Laxenaire et al. [91] studied 137 patients, reporting significant complications and the need for removal of the implant in 13.8% of patients due to infection, implant migration, cutaneous fistulas, dacryocystitis and persistent diplopia. Similarly, Aronowitz et al. [22] found short- and long-term complication rates of 3.9% and 2.8%, respectively. Therefore, despite its favourable characteristics, silicone has been disfavoured due to its complications and the development of other materials.

### 3.8. Resorbable Alloplastic Materials

#### 3.8.1. Polyglycolic Acid (PGA)

PGA is an implant material that loses its integrity at two months and is 95% resorbed at nine months [92]. Balogh et al. [24] presented 18 patients treated with PGA and found no migration of implants, well-corrected orbital volumes, and one complication of palpebral inflammation that resolved spontaneously. They also commented that the material was easy to use. Hollier et al. [32] used PGA in defects larger than 1 cm^2^ for 12 patients with a follow-up of six months. One patient developed an inflammatory reaction requiring removal of the implant, and two others developed enophthalmos. They concluded that PGA should not be used for large defects.

#### 3.8.2. Polyglactin

Polyglactin 910 is most commonly known as the suture material Vicryl, it is a resorbable synthetic material composed of lactide and glycolide acids. Vicryl mesh is the most commonly used polyglactin 910 for orbital fractures. Proponents of its use argue that as it is resorbable, layered, so it is easily cut to the appropriate thickness, is soft and pliable, and poses no risk to the tissues of the orbital apex. A study of 28 patients over a 5-year period highlights potential disadvantages of its flimsy nature, requiring up to 56 layers, and low-grade inflammation of the eyelid up to 11 months post-operatively [45].

#### 3.8.3. Polydioxanone

Polydioxanone is a synthetic biodegradable polymer that has been recommended for the repair of orbital defects 1 to 2 cm with communication to the maxillary sinus [34]; however, histological studies show a range of host responses from minimal inflammation to fragmentation and dislocation of the material, causing significant tissue reaction [93].

Iizuka et al. [34] reconstructed defects of 1 to 2 cm in size for 20 patients. They reported the material was well tolerated with no inflammatory complications. They found the most common was inferior migration of the globe, which they recommended overcorrection at time of surgery. Ten patients showed overcorrection, of which nine had transient diplopia that resolved in seven patients at 29 days.

Bauman et al. [25] reconstructed 31 orbits with polydioxanone: One patient required removal of the implant due to haematoma and diplopia; 1 patient required partial removal of the implant due to extrusion; 25 patients had postoperative diplopia with ten having ongoing diplopia 6 months post-operatively. Seven patients developed enophthalmos, of which five had defects greater than 2.5 cm. Similarly, Kontio et al. [37] performed a study on 16 patients and reported enophthalmos in 37% of patients at 36 weeks. They also found one patient developed maxillary sinusitis requiring removal of the implant and a further four patients developing fibrotic sinuses with gas (three) and fluid (one). These authors attributed enophthalmos being a result of weak resultant scarring and recommended polydioxanone not be used for orbital repair.

#### 3.8.4. Poly-L/D-Lactic Acid

Poly-L/D-lactic acid is a bioresorbable plate that leaves a stable shelf of healed bone or soft tissue after complete resorption, providing multiple advantages over permanent implants and serving as useful alternatives to orbital floor reconstruction [42,53]. Multiple studies have identified poly-L/D-lactic acid as a safe material to use for orbital reconstruction with a low complication rate of 3.4% [27,53].

Al-Shukan and Lindqist [20] compared the use of autologous bone and poly-L/D-lactic acid for orbital fractures >2cm^2^ and found no statistically significant difference in complications with enophthalmos and diplopia, the two most common complications.

Studies investigating long-term clinical and radiological findings have identified no abnormal tissue foreign body reactions in the orbit on MRI [21,27]. Al-Shukan et al. [21] states that poly-L/D-lactic acid shows adequate strength to stabilise bone segments during the critical period of bone healing. Lieger et al. [42] support this claim by finding no evidence of sagging of orbital contents on postoperative CT scans for 46 patients treated with poly-L/D-lactic acid for orbital fractures ≥1.5 cm.

A retrospective study of 94 patients with 98 orbits who had undergone repair with poly-L/D-lactic acid found not only significant improvement in symptoms of ocular mobility, diplopia, enophthalmos and infraorbital hypoesthesia but also complete resorption of biomaterial and formation of neobone on postoperative imaging [53].

It is therefore concluded that poly-L/D-lactic acid is a safe resorbable reconstructive option with a low clinical complication rate that should be considered by surgeons.

### 3.9. Complications

The type and number of complications reported in the studies is presented in Table 3.

In total, 534 postoperative complications were reported out of 3870 patients who were operated for orbital reconstruction. Most frequent complications include diplopia (22.3%, *n* = 119), enophthalmos (21.3%, *n* = 114), removal of implant (13.9%, *n* = 74), re-operation (10.5%, *n* = 56), infection (9.2%, *n* = 49), exposure/extrusion (3.7%, *n* = 20), restricted EOM (3.6%, *n* = 19) and proptosis (3.2%, *n* = 17). All other types of complications accounted for less than 2% of the total complications. Diplopia was associated with 10 out of 16 materials used in orbital reconstruction. Enophthalmos was associated with 12 implant materials.

Complications can be attributed to surgical technique, host response, and toxicity of implant material and there is an overlap between these factors [48,56]. For example, under correction or over correction are related to surgical technique rather than material. Infraorbital nerve paraesthesia and entropian have been associated with surgical technique; however, some materials are easier to use and mould into position and hence might reduce this kind of complications.

It is useful to exclude those complications due to surgical technique and present only those for which implant material plays an important role. Complications related to material include exposure/extrusion, fistula, infection, inflammation, pain, persistent/prolonged oedema, prominence, and removal of the implant due to implant-related complications [48].

Table 4 presents only those complications that are associated with implant material. The figures have limitations due to a small number of patients and should be interpreted with caution. Complications related to implant material are reported in 9 out of 16 implant materials. The highest percentage of complications were associated with silicone (17.5%, *n* = 93), followed by polyglactin (14.3%, *n* = 4), autologous bone (unspecified donor site) (11.3%, *n* = 8), dense polyethylene (5.4%, *n* = 6), titanium (2.4%, *n* = 18), Teflon (PTFE) (2.4%, *n* = 17), Polyglycolic Acid (1.3%, *n* = 1), and polydioxanone (0.1%, *n* = 1).

## 4. Discussion

Treatment of orbital wall fractures is ever-evolving and continues to be a topic of debate. All materials, when used appropriately, produce satisfactory results. However, there is no one material universally successful. The result of this review shows that there are several easily available and user-friendly materials that provide reliable outcomes for the treatment of orbital fractures.

Autologous implants are a lifelong, reliable, biocompatible material that is still considered the gold standard but is associated with donor site morbidity. Use and morbidity often depend on the experience of the surgeon. Allogenic materials are resorbable but consideration of potentially a severe disease transmission must be given. Alloplastic materials are gaining popularity due to their ease of use, availability and reduced surgical morbidity; however, permanent alloplastic materials are associated with risks of a permanent foreign body, while resorbable materials fail to form adequate bone and resultant scarring is too weak to support the orbit’s contents.

The success of material used in orbital floor fractures is determined by various factors, including patient selection, the timing of surgery, execution of sound surgical principles, and the type of implant material used. Selection of implant material is largely dependent upon the surgeon’s preference and the chosen surgical technique. There are currently very few studies exclusively comparing the selection of reconstructive materials for orbital floor fractures. Therefore, further research should prospectively compare commonly used materials while standardising other factors to allow a direct comparison of reconstructive materials. This would help develop guidelines on the selection of implant material for treating orbital fractures.

## 5. Conclusions

The ideal material is one that is resorbable, osteoconductive, resistant to infection, minimally reactive, does not induce capsule formation, allows for bony ingrowth, cheap, and readily available. This article highlights that an appropriate knowledge of materials is critical and appropriate use as well as patient selection will reduce the risk of complications. In future, it could be helpful conducting a prospective study comparing the most commonly used materials by similar surgical units with surgeons matched to experience. This might highlight superiority of one material over another.

## Figures and Tables

**Table 1:** American Society of Plastic Surgeons Level of Evidence Rating Scale for Therapeutic Studies.

Level of Evidence	Qualifying Studies
I	High-quality, multi-centered or single-centered, randomized controlled trial with adequate power; or systematic review of these studies
II	Lesser-quality, randomized controlled trial; prospective cohort or comparative study; or systematic review of these studies
III	Retrospective cohort or comparative study; case-control study; or systematic review of these studies
IV	Case series with pre/post test; or only post test
V	Expert opinion developed via consensus process; case report or clinical example; or evidence based on physiology, bench research or “first principles”

**Table 2:** List of retrieved studies reported on implant material and postoperative complications.

Author	Level of Study	Type of Study	Implant Materials	Follow-Up Period
Aitasalo et al. [19]	III	Retrospective review	Bioactive glass	1 to 12 months
Al-Sukhun and Lindqvist [20]	III	Comparative study	Autogenous bone grafts, poly-L/DL-Lactide [P(L/DL)LA 70/30]	2–36 weeks
Al-Sukhun et al. [21]	III	Retrospective cohort study	Poly-L/DL-Lactide [P(L/DL)LA 70/30]	-
Aronowitz et al. [22]	III	Retrospective study	Teflon (PTFE)	Mean, 16 years
Asamura et al. [23]	III	Retrospective cohort study	Ilium and periosteum polymer complex	2–22 months
Balogh et al. [24]	III	Retrospective cohort study	Polyglycolic acid (PGA)	24 to 43 months
Baumann et al. [25]	III	Follow-up study	Polydioxanone	6 months
Brucoli et al. [5]	III	Retrospective cohort study	Autologous calvarial bone, titanium mesh, tutopatch sheet	Mean, 39 months
Constantian [26]	IV	Case series	Autogenous tissues	7 months to 3.5 years
Cordewener et al. [27]	III	Retrospective cohort study	Poly-D-Lactic Acid	-
Düzgün and Sirkeci [28]	III	Comparative study	Cartilage, bone grafts, titanium mesh, porous polyethylene implant	Mean, 14 months
Ellis and Tan [29]	III	Retrospective review	Cranial bone grafts, titanium mesh	-
Guerra et al. [30]	III	Retrospective study	Allogenic lyophilized dura	3 months to 1 year
Guo et al. [31]	III	Comparative study	Calvaria bone graft, titanium mesh	>2 weeks
Hollier et al. [32]	III	Retrospective cohort study	Polyglycolic Acid (PGA)	Upto 12 months
Holtmann et al. [17]	III	Retrospective study	Titanium mesh	-
Hwang and Kita [33]	III	Prospective study	Titanium mesh	-
Iizuka et al. [34]	III	Prospective study	Polydioxanone	9 to 45 months
Kinnunen et al. [35]	III	Comparative study	Autogenous ear cartilage, bioactive glass	2 to 5 years
Kirby et al. [6]	III	Retrospective cohort study	Autologous bone, Titanium, porous polyethylene	Mean, 38.8 weeks
Klisovic et al. [36]	IV	Case report	Silicone	18 months
Kontio et al. [37]	III	Prospective study	Polydioxanone	Mean, 29 weeks
Kontio et al. [38]	III	Prospective study	Iliac bone graft	Mean, 7.8 months
Kraus et al. [39]	III	Prospective study	Autogenous septal cartilage	1 week to 6 months
Lai, A. [40]	III	Prospective study	Nasal septal cartilage	3 months to 4 years
Lee and Nunery [41]	III	Retrospective review	Titanium mesh and titanium plate	5 to 18 months
Lieger et al. [42]	III	Retrospective study	Poly-L/DL-Lactide [P(L/DL)LA 70/30]	3 to 12 months
Lipshutz and Ardizone [43]	IV	Case series	Silicone	-
Lupi et al. [44]	III	Retrospective study	Porous polyethylene	-
Mauriello et al. [45]	III	Case series	Polyglactin	1 to 24 months
Polley and Ringler [46]	III	Retrospective study	Teflon (PTFE)	3 months to 15 years
Romano et al. [47]	III	Prospective study	Porous polyethylene	-
Rubin and Yaremchuk [48]	III	Comprehensive review	Porous polyethylene, dense polyethylene, silicone, tefflon (PTFE)	-
Sewall et al. [49]	IV	Case report	Silicone	-
Sugar et al. [50]	III	Prospective study	Titanium mesh	Mean, 24 months
Waite and Clanton [51]	III	Prospective study	Lyophilized dura	12 months
Wang et al. [52]	III	Retrospective study	Autogenous bone, titanium mesh, Medpor	1 to 6 months
Young et al. [53]	III	Retrospective review	Poly-L/DL-lactide (P[L/DL]LA) 85/15, (P[L/DL]LA) 70/30, Polycaprolactone	15 to 24 months
Zunz et al. [54]	IV	Case series	Calvarial, iliac autogenous bone grafts	Mean, 12.5 months

- Not reported.

**Table 3:** Reconstruction materials and postoperative complications.

Implant Material	Author	Patients	Complications	Total Complications
Autologous Calvarial Bone	Brucoli et al. [5] Guo et al. [31] Ellis and Tan [29] Zunz et al. [54]	87	Diplopia (7) Enophthalmos (10)	17
Autologous Iliac Bone	Düzgün and Sirkeci [28] Zunz et al. [54] Asamura et al. [23] Al-Sukhun and Lindqvist [20] Kontio et al. [38]	72	Diplopia (15) Haematoma donor site (2) Enophthalmos (4) Infraorbital nerve paraesthesia (2) Orbital dystopia (7)	30
Autologous Bone (unspecified donor site)	Kirby et al. [6]	71	Re-operation (17) Removal (4) Diplopia (10) Enophthalmos (16) Restricted EOM (2) Infection (4) Proptosis (4)	57
Autologous Conchal Ear Cartilage	Constantian [26] Kinnunen et al. [35] Düzgün and Sirkeci [28]	33	Diplopia (8) Enophthalmos (2) Infraorbital nerve paraesthesia (2)	12
Autologous Nasal Septal Cartilage	Kraus et al. [39] Lai et al. [40]	33	Enophthalmos (1) Infraorbital nerve paraesthesia (2) Lower lid oedema (1)	4
Allogenic Lyophilized Dura	Waite and Clanton [51] Guerra et al. [30]	70	Enophthalmos (3) Infraorbital paraesthesia (4) Cicatricial problems (2)	9
Titanium	Brucoli et al. [5] Kirby et al. [6] Holtman et al. [17] Sugar et al. [50] Hwang and Kita [33] Ellis and Tan [29] Lee and Nunery [41] Düzgün and Sirkeci [28]	741	Re-operation (16) Removal (10) Diplopia (14) Enophthalmos (12) Restricted EOM (15) Infection (8) Proptosis (5)	80
Porous Polyethylene	Kirby et al. [6] Rubin and Yaremchuk [48] Romano et al. [47] Hwang and Kita [33] Düzgün and Sirkeci [28] Lupi et al. [44]	326	Re-operation (23) Removal (7) Diplopia (9) Enophthalmos (13) Restricted EOM (2) Infection (7) Proptosis (8) Overcorrection (1) Undercorrection (1) Implant extrusion (2)	73
Dense Polyethylene	Rubin and Yaremchuk [48]	78	Removal (1) Infection (2) Oedema (3)	6
Bioactive Glass	Kinnunen et al. [35] Aitasalo et al. [19]	50	Diplopia (5) Infraorbital nerve dysfunction (6) Entropion (1) Removal (1)	13
Silicone	Rubin and Yaremchuk [48] Sewall et al. [49] Hwang and Kita [33] Lipshutz and Ardizone [43] Klisovic et al. [36]	530	Infection (25) Exposure/extrusion (16) Persistent oedema (2) Prominence (2) Pain (6) Removal (42)	93
Teflon (PTFE)	Rubin and Yaremchuk [48] Polley and Ringler [46] Aronowitz et al. [22]	702	Diplopia (11) Enophthalmos (15) Infection (3) Exposure/extrusion (4) Removal (9) Fistula (1)	43
Polyglycolic Acid (PGA)	Balogh et al. [24] Hollier et al. [32]	78	Enophthalmos (2) Inflammatory reaction (1) Inflammation (1)	4
Polyglactin	Mauriello et al. [45]	28	Inflammation (4)	4
Polydioxanone	Holtman et al. [17] Kontio et al. [37] Iizuka et al. [34] Baumann et al. [25]	774	Diplopia (38) Exophthalmos (10) Enophthalmos (29) Prolonged oedema (1)	78
Poly-D-Lactic Acid	Cordewener et al. [27] Al-Sukhun et al. [21] Al-Sukhun and Lindqvist [20] Leiger et al. [42] Young et al. [53]	176	Diplopia (1) Exophthalmos (5)	6
Unspecified Implants	Wang et al. [52]	21	Diplopia (1) Enophthalmos (2) Infraorbital numbness (2)	5

**Table 4:** Postoperative complications attributed to implant material.

Implant Material	Total Number of Patients	Complications Rates (%)								
		Exposure/Extrusion	Fistula	Infection	Inflammation	Pain	Persistent/Prolonged Oedema	Prominence	Removal of Implant	Overall
Autologous Calvarial Bone	87	0	0	0	0	0	0	0	0	0
Autologous Iliac Bone	72	0	0	0	0	0	0	0	0	0
Autologous Bone (unspecified donor site)	71	0	0	5.6	0	0	0	0	5.6	11.3
Autologous Conchal Ear Cartilage	33	0	0	0	0	0	0	0	0	0
Autologous Nasal Septal Cartilage	33	0	0	0	0	0	0	0	0	0
Allogenic Lyophilized Dura	70	0	0	0	0	0	0	0	0	0
Titanium	741	0	0	1.1	0	0	0	0	1.3	2.4
Porous Polyethylene	326	0.7	0	2.3	0	0	0	0	2.3	5.4
Dense Polyethylene	78	0	0	2.6	0	0	3.8	0	1.3	7.7
Bioactive Glass	50	0	0	0	0	0	0	0	2.0	2.0
Silicone	530	3.0	0	4.7	0	1.1	0.4	0.4	7.9	17.5
Teflon (PTFE)	702	0.6	0.1	0.4	0	0	0	0	1.3	2.4
Polyglycolic Acid (PGA)	78	0	0	0	1.3	0	0	0	0	1.3
Polyglactin	28	0	0	0	14.3	0	0	0	0	14.3
Polydioxanone	774	0	0	0	0	0	0.1	0	0	0.1
Poly-D-Lactic Acid	176	0	0	0	0	0	0	0	0	0
Unspecified Implants	21	0	0	0	0	0	0	0	0	0
